# Acute Management Should Be Optimized in Patients with Less Specific Stroke Symptoms: Findings from a Retrospective Observational Study

**DOI:** 10.3390/jcm10051143

**Published:** 2021-03-09

**Authors:** Simona Halúsková, Roman Herzig, Dagmar Krajíčková, Abduljabar Hamza, Antonín Krajina, Vendelín Chovanec, Miroslav Lojík, Jan Raupach, Ondřej Renc, Libor Šimůnek, Eva Vítková, Lukáš Sobíšek, Martin Vališ

**Affiliations:** 1 Department of Neurology, Comprehensive Stroke Center, Charles University Faculty of Medicine and University Hospital, Sokolská 581, CZ-500 05 Hradec Králové, Czech Republic; sim.haluskova@gmail.com (S.H.); dagmar.krajickova@fnhk.cz (D.K.); libor.simunek@email.cz (L.Š.); eva@kinoaero.cz (E.V.); lukas.sobisek@yahoo.com (L.S.); valismar@seznam.cz (M.V.); 2 Department of Neurology, Charles University Faculty of Medicine, CZ-500 03 Hradec Králové, Czech Republic; hamzaabdulj@gmail.com; 3 Department of Radiology, Comprehensive Stroke Center, Charles University Faculty of Medicine and University Hospital, CZ-500 05 Hradec Králové, Czech Republic; antonin.krajina@fnhk.cz (A.K.); chovanec.v@seznam.cz (V.C.); miroslav.lojik@fnhk.cz (M.L.); jan.raupach@fnhk.cz (J.R.); ondrej.renc@fnhk.cz (O.R.)

**Keywords:** acute ischemic stroke, clinical symptoms, intravenous thrombolysis, endovascular therapy, recanalization times, clinical outcome

## Abstract

Anterior circulation stroke (ACS) is associated with typical symptoms, while posterior circulation stroke (PCS) may cause a wide spectrum of less specific symptoms. We aim to assess the correlation between the initial presentation of acute ischemic stroke (AIS) symptoms and the treatment timeline. Using a retrospective, observational, single-center study, the set consists of 809 AIS patients treated with intravenous thrombolysis (IVT) and/or endovascular treatment (EVT). We investigate the impact of baseline clinical AIS symptoms and the affected vascular territory on recanalization times in patients treated with IVT only and EVT (±IVT). Regarding the IVT-only group, increasing the National Institutes of Health Stroke Scale (NIHSS) score on admission and speech difficulties are associated with shorter (by 1.59 ± 0.76 min per every one-point increase; *p* = 0.036, and by 24.56 ± 8.42 min; *p* = 0.004, respectively) and nausea/vomiting with longer (by 43.72 ± 13.13 min; *p* = 0.001) onset-to-needle times, and vertigo with longer (by 8.58 ± 3.84 min; *p* = 0.026) door-to-needle times (DNT). Regarding the EVT (±IVT) group, coma is associated with longer (by 22.68 ± 6.05 min; *p* = 0.0002) DNT, anterior circulation stroke with shorter (by 47.32 ± 16.89 min; *p* = 0.005) onset-to-groin time, and drooping of the mouth corner with shorter (by 20.79 ± 6.02 min; *p* = 0.0006) door-to-groin time. Our results demonstrate that treatment is initiated later in strokes with less specific symptoms than in strokes with typical symptoms.

## 1. Introduction

Acute ischemic stroke (AIS) typically presents with the sudden onset of neurological deficit. Clinical manifestation depends mainly on the AIS localization and the volume of the affected brain tissue, which are associated with the involved vascular territory. Occlusion of the internal carotid arteries (ICA), middle cerebral arteries (MCA), and of the anterior cerebral arteries (ACA) or their branches results in anterior circulation stroke (ACS), accounting for approximately 70–80% of all AIS. Posterior circulation stroke (PCS) refers to any infarction localized in the regions supplied by the vertebrobasilar arterial system with reported prevalence ranging from 20 to 30% [1,2,3,4]. Symptoms of ACS include contralateral hemiparesis and/or hemianesthesia, central facial palsy, forced gaze deviation toward the lesion site, dysarthria, aphasia (dominant hemisphere), and neglect syndrome (non-dominant hemisphere). To contrast to ACS, PCS causes a wide spectrum of less specific symptoms, such as vertigo, headache, nausea and vomiting, diplopia, visual field disturbances, slurred speech, gait and limb ataxia, or alteration of consciousness [3,5].

Although the most common symptoms of ACS and PCS are well described, reliable differentiation between ACS and PCS can be challenging. How the stroke symptoms are described and how a patient presents at the emergency room affects the delay between stroke onset and the start of treatment. Several randomized controlled trials demonstrated that both intravenous thrombolysis (IVT) with the administration of recombinant tissue plasminogen activator (rtPA) and endovascular therapy (EVT) were highly time-sensitive treatments—the earlier they are commenced the better is the chance for achieving a favorable outcome [6,7]. Nevertheless, only a few studies assessed the impact of specific AIS symptoms on the recanalization times within the limited treatment window [8,9].

Our aim is to investigate the impact of initial presentation of AIS symptoms and the affected vascular territory on recanalization times in patients treated with IVT only and with EVT (±IVT).

## 2. Materials and Methods

### 2.1. Data Source and Study Population

All relevant data used for this retrospective analysis were manually extracted from hospital information systems and available individual patient medical charts, including documentation from the referring hospital (in the case of patients with secondary transport), emergency physician notes, neurology notes, and medication administration records.

During a retrospective, observational, single-center study, prospectively collected data of 809 consecutive AIS patients aged ≥18 years and treated with IVT only or EVT (±IVT) between 1 January 2013 and 31 December 2018 were analyzed. All EVT procedures were performed at the Comprehensive Stroke Center (CSC), Hradec Králové, Czech Republic. Ninety-eight patients from the EVT group received IVT in the primary stroke centers (PSC) according to the geographic area and then were subsequently transferred to our CSC. Patients experiencing in-hospital stroke (38) also were enrolled in this analysis. Each stroke was considered an independent event, regardless of whether it was the first hospital stay or a readmission. Patients were considered eligible for the analysis if data about the involvement of the particular territory (ACS or PCS) were available. Patients with an unclear stroke territory (e.g., thalamic infarcts or border zone infarcts in the posterior cerebral artery (PCA)/MCA watershed) or AIS involving both anterior and posterior circulation were excluded in the data collection phase already. ACS was classified as symptomatic ischemia involving the ICA, MCA, or ACA territories. PCS was defined as symptomatic ischemia occurring within the territory of the vertebral artery (VA), basilar artery (BA), or PCA. No additional exclusion criteria were applied. Initial routine investigation in the emergency room comprised neurological, physical, and laboratory examinations and assessment of the admission neurological deficit using the National Institutes of Health Stroke Scale (NIHSS) [10] performed by a certified neurologist.

### 2.2. Neuroimaging

All patients underwent the standardized stroke imaging protocol for the assessment of the eligibility for IVT and endovascular treatment EVT, as described in detail previously [11]. This protocol included non-enhanced computed tomography (CT) of the brain with the assessment of the Alberta Stroke Program Early CT Score (ASPECTS) and CT angiography (CTA) of the cervical and intracranial arteries. Patients treated beyond 6 h after the onset of the first symptoms, or with an unknown time of stroke onset, also underwent a perfusion CT scan [12]. Regarding patients needing secondary transport to the CSC and with preceding IVT administration in the PSC, a non-enhanced brain CT control was performed in the CSC prior to an intended EVT to exclude IVT-related hemorrhagic complications or the development of an extensive brain infarction only if the transport took more than one hour, and/or the patient’s neurological status deteriorated significantly.

### 2.3. Recanalization Treatment

Recanalization treatment was performed in agreement with the valid national and international guidelines [13,14,15,16,17]. IVT with a standard dose of 0.9 mg/kg (maximum dose of 90 mg) of rtPA (Actilyse^®^; Boehringer Ingelheim, Ingelheim am Rhein, Germany) was administered within 4.5 h from the “last-known-well” condition, with 10% of the dose given as an intravenous initial bolus and the remaining 90% of the dose as a 60-min infusion in all patients fulfilling the inclusion and exclusion criteria.

A mechanical thrombectomy (MT) using stent-retrievers was started as soon as possible, without waiting for the effect of the IVT (if applied) and within a standard 6-h time window from AIS symptom onset in ACS patients with an ASPECTS ≥ 6 on a non-enhanced brain CT. Regarding ACS patients treated more than 6 h after AIS symptom onset, or with an unknown AIS onset time, a MT was indicated based on the perfusion CT results—it was performed in patients with a small ischemic core (≤70 mL) [18] and with the presence of ischemic penumbra. Regarding patients with PCS due to BA occlusion, a MT was performed within a 24-h time window in the case of the absence of an extensive brain infarction. The choice of the particular stent-retriever used for clot extraction was at the discretion of the treating interventional neuroradiologist. Concerning most patients, a MT was performed under conscious sedation and general anesthesia was avoided whenever possible after evaluation by a dedicated anesthesiology team.

Concerning patients with concurrent ICA occlusion (so called “tandem occlusion”), carotid artery stenting was performed under local anesthesia using a standard catheterization approach from the femoral artery via an 8F or 9F sheath introduced into the common carotid artery. During most procedures a self-expandable carotid stent was implanted after predilatation using a low profile balloon as the first step, followed by a MT using a balloon-guiding catheter placed in the ICA above the level of the carotid stent.

### 2.4. Observed Parameters

The following parameters were observed in both the IVT only and EVT (±IVT) groups: patient age and sex, baseline neurological deficit (assessed using the NIHSS score), involved vascular territory (anterior/posterior), and the presence of nine selected clinical symptoms—limb weakness (mono- or hemiparesis/hemiplegia; HEMIPAR), facial palsy (drooping of the corner of the mouth; N VII), speech difficulties (dysarthria/phatic disorder; SPEECH), sensory impairment (hypoesthesia/anesthesia/paresthesias; SENSATION), visual disturbances (diplopia/visual field defects; VISION), vertigo (VERTIGO), headache (HEADACHE), nausea/vomiting (VOMIT), and loss of consciousness (COMA). Regarding patients with a previous stroke, only the occurrence of new symptoms or a clear progression of possible residual symptoms were included in the analysis. Concerning the EVT group, we additionally evaluated the use of IVT before the EVT and localization of the arterial occlusion—in the extracranial ICA (ICAe), intracranial ICA (ICAi)*,* M1 segment of the MCA (MCA/M1), M2 segment of the MCA (MCA/M2), ACA, PCA, VA, or BA.

Regarding both groups, times of symptoms onset, times of arrival to the emergency department in our hospital, times of IVT bolus dose administration and, in the EVT (±IVT) group, arterial puncture times also were recorded. Based on these times, five time intervals were evaluated—onset-to-door time (ODT), onset-to-needle time (ONT) and door-to-needle time (DNT) in both groups and, onset-to-groin time (OGT) and door-to-groin puncture time (DGT) in the EVT (±IVT) group. Regarding patients with an unknown time of stroke onset, only the time intervals after their arrival to the hospital were analyzed.

### 2.5. Statistical Analysis

The IVT only and EVT (±IVT) groups were compared using a chi-square test of independence for categorical variables (sex, clinical symptoms occurrence, vascular territory). Group differences in medians were compared by a non-parametric Mann-Whitney U test with non-pooled SDs for numeric parameters like time intervals, the NIHSS, and age. The Benjamini-Hochberg procedure was used to minimize the false discovery rate. Regarding the IVT only group, we investigated whether there was a significant relationship between the key time intervals (ODT, ONT, DNT) and the independent variables (age, sex, admission NIHSS, involved vascular territory, and initial presenting symptoms of AIS). Regarding the EVT (±IVT) group, we determined five outcome time intervals (ODT, ONT, DNT, OGT, DGT) and we aimed to assess the relationship between those time points and specific independent variables (age, sex, admission NIHSS, involved vascular territory, initial clinical symptoms, use of IVT, and localization of the occlusion in particular arteries). The time-interval outcomes were log-transformed for regression modelling because they were positively skewed. The series of univariate linear regression models in both groups were fitted for logarithmized time intervals to identify the dependency on each parameter (explanatory variable). To find a combination of explanatory variables that were able to describe the dependent variable more precisely, we next used a multivariable linear model. The suitable combinations of explanatory variables were detected by two procedures—Stepwise selection (implemented in R package MASS) and by the “leapBackward” cross-validated (5-folds) method from package leaps. The best multivariable model was finally chosen according to three information criteria: adjusted R2 (index of determination), PRESS (predicted residual error sum of squares) and RMSE (Residual Mean Square Error). All analyses were performed using the statistical software R (www.r-project.org/ (accessed on 9 March 2021).) version 3.5.3; the reported *p* values were two-tailed and a 5% significance level was chosen.

### 2.6. Ethics

The entire study was conducted in accordance with the Declaration of Helsinki of 1964 and its later amendments (including the last in 2013). All procedures were performed in accordance with institutional guidelines. The study was approved by the Ethics Committee of the University Hospital Hradec Králové (approval No. 202005 S05P). All conscious patients signed informed consent forms for the eligible and available diagnostics and treatment. Independent witnesses verified the signatures in cases in which there were technical problems.

## 3. Results

**Figure 1:**
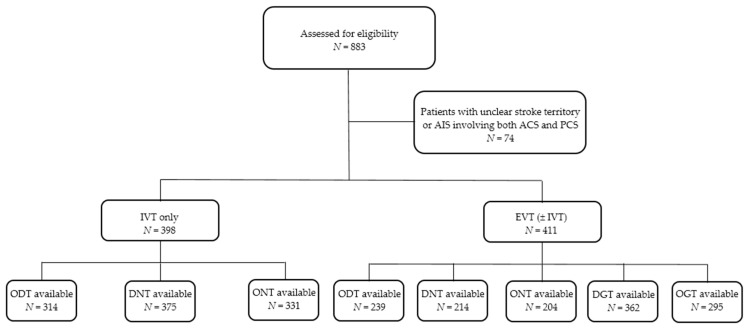
Flowchart of the study. ACS, anterior circulation stroke; AIS, acute ischemic stroke; DGT, door-to-groin puncture time; DNT, door-to-needle time; EVT, endovascular therapy; IVT, intravenous thrombolysis; *N*, Number; ODT, onset-to-door time; OGT, onset-to-groin time; ONT, onset-to-needle time; PCS, posterior circulation stroke.

Out of 809 enrolled consecutive AIS patients, 398 (49.2%) patients were treated with IVT only and 411 (50.8%) with EVT (±IVT). The baseline characteristics of the study population are shown in Table 1. The majority (74.9%) of patients had isolated large vessel occlusion. Nevertheless, in some patients, occlusion of several arteries was present. The most common occlusion site was the MCA/M1 (found in 71.8% of patients), followed by the ICAi (15.1%), MCA/M2 (13.1%), ICAe (11.2%), BA (8.3%), PCA (2.9%), VA (2.7%) and ACA (1.9%). Tandem pathology (defined as ICA+MCA M1/M2 occlusion) was detected in 11.2% of patients. The symptoms of SENSATION, VISION, VERTIGO, HEADACHE, VOMIT, and COMA were significantly more frequent in the IVT only group, whereas symptoms HEMIPAR and N VII occurred significantly more often in the EVT (±IVT) group. Clinical symptoms HEMIPAR, N VII and SPEECH were significantly more frequent in patients with ACS, while symptoms VISION, VERTIGO, HEADACHE, VOMIT and COMA were detected more often in patients diagnosed with PCS (statistical evaluation was not possible in the case of the last three mentioned symptoms due to their minimal occurrence in the ACS group) (Table 2). Observed time intervals were available for the following numbers of patients in the particular groups: ODT in 314 (78.9%) and in 239 (58.2%), ONT in 331 (82.2%) and in 204 (49.6%), respectively, DNT in 375 (94.2%) and in 214 (52.1%). Regarding the EVT (±IVT) group, OGT values were available in 295 (71.8%) and DGT in 362 (88.1%) patients (Figure 1). ONT and DNT were significantly longer (approximately by 18 and 9 min, respectively) in the IVT only group.

Table 3 shows the results of the univariate regression analysis assessing the dependency of recanalization times on the observed parameters both in the IVT only and the EVT (±IVT) groups. The results of the multivariable linear model for recanalization times are presented in Table 4. Regarding the IVT only group, the ODT was best described by a combination of four variables—the baseline NIHSS score (every one-point increase in the NIHSS value is expected to shorten the ODT by 1.74 min) and the presence of clinical symptoms SPEECH (ODT shortening by 18.9 min), HEMIPAR (ODT shortening by 12.6 min) and VOMIT (ODT prolongation by 31.2 min). Similarly, the ONT was best characterized by a combination of four factors—admission NIHSS (every one-point increase in the NIHSS value is expected to shorten the ONT by 1.59 min) and the presence of clinical symptoms SPEECH (ONT shortening by 24.6 min), HEMIPAR (ONT shortening by 15.1 min) and VOMIT (ONT prolongation by 43.7 min). The clinical symptoms SPEECH and VERTIGO were defined as the best variables of DNT in IVT-treated patients, with DNT shortening by 5.2 min and prolongation by 8.6 min, respectively. Concerning the EVT (±IVT) group, no significant ODT predictor was identified. Affected anterior circulation and the presence of clinical symptom N VII had the highest predictive value for the ONT (with shortening by 24.8 and 15.8 min, respectively). Concerning the same group, only one explanatory variable was sufficient to accurately describe the remaining time intervals—presence of symptom COMA for the DNT (prolongation by 22.7 min), affected the anterior vascular territory for the OGT (shortening by 47.3 min) and presence of symptom N VII for the DGT (shortening by 20.8 min).

## 4. Discussion

While there are many studies investigating the impact of the severity of the neurological deficit assessed by the NIHSS [19,20,21,22,23] or AIS localization (PCS versus ACS) [8,19,24] on specific treatment time intervals, studies focusing on individual stroke symptoms are rare [8,9]. According to our knowledge, our study is just the second original research manuscript dealing with this topic, as the study performed by Baraban et al. was published in the form of an abstract only [9].

During this study we identified several variables (the NIHSS value, anterior circulation, clinical symptoms HEMIPAR, N VII, SPEECH, VERTIGO, VOMIT, COMA) of five observed time intervals. Aligned with the previous literature, we observed that presenting symptoms did impact the treatment timeline [8,9,25]. Regarding AIS presenting with less specific symptoms (VERTIGO, VOMIT, COMA), the treatment was initiated later than in AIS with more defined clinical symptoms (HEMIPAR, N VII, SPEECH) related mainly to ACS. Since nonspecific symptoms such as VERTIGO and VOMIT, commonly occurring in PCS, overlap with more benign medical conditions like gastroenteritis, we assume that these patients will not have ONT, OGT, DNT, DGT only, but also ODT due to an inappropriate response (delay in calling 911 by patient/relative/friend). This hypothesis was confirmed by Baraban et al. who found that patients presenting with symptom SPEECH arrived at the hospital 14.2% faster (*p* = 0.007) and also had a 6.0% faster DNT (*p* = 0.006) than patients without these symptoms. Moreover, authors observed that those presenting with HEMIPAR had a 9.3% faster DNT (*p* = 0.001) and patients with other neurologic symptoms arrived 14.0% later than those without HEMIPAR (*p* = 0.009) [9]. According to Sarraj et al. [8], ONT was statistically different for the following clinical symptoms: N VII (153 versus 167 min in the case of its absence, *p* = 0.044), VOMIT (187 versus 156 min, *p* = 0.009), and COMA (153 versus 171 min, *p* = 0.006). DNT was significantly associated with HEMIPAR (74 versus 87 min in the case of its absence, *p* = 0.014) and VOMIT (96 versus 75 min, *p* = 0.005) [8]. Authors from Finland similarly demonstrated that AIS patients with a positive Face Arm Speech Time (FAST) test had a shorter DNT as well (48 versus 66 min, *p* < 0.001) [25]. Interestingly, in contrast to some previous reports in which HEMIPAR was associated with reduced DNT [8,9], in our study the presence of this symptom only insignificantly influenced ODT and ONT, even though it seems to be an easily recognizable stroke symptom. Conversely, the presence of the clinical symptom SPEECH was associated with a shorter ODT, ONT, and DNT in our IVT only group, and the presence of clinical symptom N VII was associated with a shorter ONT and DGT in our EVT (±IVT) group. The SPEECH symptom included not only dysarthria, but also phatic disorder, which represents a more remarkable neurological deficit. The symptom N VII, although representing a “minor stroke symptom”, is easily recognizable by the public, similar to HEMIPAR. Even an isolated phatic disorder can justify the IVT procedure, as it might be associated with up to four points on the NIHSS, i.e., the cut-off point for rtPA administration) [13,14,15,16,17]. Although significant variables for the observed time intervals were slightly different in our and the above-mentioned studies, it was confirmed that patients with less specific symptoms experienced treatment delays. The symptom COMA represented the only exception—it was responsible for DNT prolongation in our EVT (±IVT) group by 22.7 min, but in the study by Sarraj et al., COMA significantly shortened ODT by approximately 12 min and ONT by approximately 18 min [8]. One possible interpretation could be the difference in the pre-hospital and intra-hospital management of comatose patients. Since COMA is a serious medical condition usually recognizable by the general public, the patient probably gets to the hospital quickly. However, the problem may be the intra-hospital delays due to the need for a systematic multidisciplinary approach to the unconscious patient, e.g., early physiological stabilization, activation of an anesthesia team, intubation for respiratory failure or airway protection [26]. Hassan et al. found that the mean time interval between the CT scan and the initiation of an endovascular procedure was significantly longer in patients who underwent preprocedural intubation (132 ± 102 versus 111 ± 47 min, *p* < 0.0001) [27]. Ultimately, our findings reflect a real clinical practice. There are several explanations of this phenomenon. Screening tools such as the FAST test developed for prehospital identification of AIS patients by checking for facial and/or arm weakness and speech disturbance are undoubtedly less sensitive for the identification of PCS compared to ACS [24,28,29]. Since symptoms of PCS can mimic other disorders, they can be misinterpreted easily and may lead to under-recognition considering initial nonfocal and nonspecific symptoms [24,30,31]. Both ONT and DNT were significantly longer in the PCS versus the ACS group (175 versus 155 min, *p* = 0.0121 and 90 versus 74 min, *p* = 0.0026, respectively) in the study published by Sarraj et al. [8]. Likewise, DNT was, on average, longer by 13 min in patients with PCS compared to ACS patients (*p* < 0.001) according to data reported by Sommer et al. [24]. During the Czech study which aimed to determine the predictors of calling 911 in reaction to stroke symptoms, responders identified SPEECH (37%) and HEMIPAR (34%) as the most typical symptoms of AIS [32]. A Swedish study reported that two-thirds of the population knew at least one stroke symptom, but only one-tenth knew three stroke symptoms [33]. Although public awareness of stroke has improved in recent years thanks to various mass media intervention campaigns advertising AIS symptoms, there is still a lack of recognition of mainly PCS symptoms, which implies a need for increased education not only of the general public, but also of paramedics and staff working in the emergency departments. It is obvious that the initial assessment phase is crucial, and better clinical recognition is urgently needed to optimize acute care. Even patients with less specific stroke symptoms must be promptly diagnosed and treated, although this still represents a challenge in emergency medicine. Last but not least, we found that every one-point increase in the admission NIHSS value was expected to shorten ONT by 1.59 min in the IVT only group. Most authors previously demonstrated that lower baseline NIHSS scores in AIS patients were associated with longer treatment times [20,23,34]. This fact is related to the above-discussed issue concerning an often difficult differential diagnosis of PCS. Since the NIHSS is weighted more toward ACS symptoms, it tends to underestimate the clinical severity in PCS, which is reflected by previous observations of overall lower NIHSS scores in patients with PCS compared to ACS [8,24,35,36]. During our study, “more defined” stroke symptoms, such as HEMIPAR, N VII and SPEECH were more frequent in the ACS, while in the PCS, “less defined” stroke symptoms, such as VISION, VERTIGO, HEADACHE, VOMIT and COMA were observed. However, ACS of low severity is also difficult to diagnose. Thus, this problem is not restricted to PCS.

Several limitations of the present study should be mentioned. First, it has a retrospective character with a sample extracted from a single stroke center database; therefore, the results may not be generalizable. Second, the data collection methods among databases may be the source of selection bias. Our study has the same limits as all non-randomized controlled trials. Third, reported data depend on the accuracy and the completeness of the medical records. Unfortunately, data missingness was present for some time outcomes, as mentioned above. We focused on five selected time intervals including onset-to-treatment, although the time of the stroke onset is often inaccurate. Actually, some strokes may have occurred during sleep and, in other cases, the witnesses were unable to recall the precise time of symptom onset. Conversely, the strength of this study is that it included a relatively large cohort of patients. We should note that, unlike other studies, we also evaluated the clinical profile behind the NIHSS values.

## 5. Conclusions

Our results demonstrated that initial presenting symptoms of AIS did influence the treatment timeline both in the pre-hospital and intra-hospital management phase. Patients with less specific stroke symptoms associated with posterior circulation experienced treatment delays. There is no doubt that the significant diagnostic ambiguity of PCS represents a serious issue in the field of emergency medicine. Therefore, an important research question for stroke specialists still remains regarding optimized logistics and acute phase management strategies. Analysis of a larger nationwide registry and of international registries would be beneficial to confirm our observations.

## Figures and Tables

**Table 1:** Baseline and outcome characteristics.

Characteristic	IVT Only Group	EVT (±IVT) Group	*p*
*N*	398 (49.2)	411 (50.8)	N/A
Age, (years) †	71.17 ± 12.75 (72.0)	71.99 ± 12.43 (74.0)	0.2354
Male sex	223 (56.0)	172 (41.8)	0.0001
NIHSS baseline †	7.68 ± 5.00 (6.0)	14.18 ± 6.04 (14.0)	<0.0001
Vascular territory			0.7798
Anterior	346 (86.9)	361 (87.8)	
Posterior	52 (13.1)	50 (12.2)	
Clinical symptoms			
Limb weakness (HEMIPAR)	314 (78.9)	388 (94.4)	<0.0001
Drooping of the mouth corner (N VII)	283 (71.1)	342 (83.2)	0.0001
Speech difficulties (SPEECH)	313 (78.6)	341 (83.0)	0.1582
Sensory impairment (SENSATION)	61 (15.3)	18 (4.4)	<0.0001
Visual problems (VISION)	28 (7.0)	14 (3.4)	0.0363
VERTIGO	37 (9.3)	9 (2.2)	0.0001
HEADACHE	16 (4.0)	2 (0.5)	0.0020
Nausea and/or vomiting (VOMIT)	29 (7.3)	9 (2.2)	0.0015
Loss of consciousness (COMA)	3 (7.5)	26 (6.3)	0.0001
IVT	398 (100.0)	253 (61.6)	<0.0001
Time intervals (min)			
Onset-to-door (ODT) †	97.14 ± 57.35 (80.5)	105.30 ± 70.82 (85.0)	0.4089
Onset-to-needle (ONT) †	143.57 ± 64.99 (135.0)	125.28 ± 45.51 (119.0)	0.0005
Door-to-needle (DNT) †	50.10 ± 21.70 (47.0)	41.17 ± 17.29 (40.0)	<0.0001
Onset-to-groin (OGT) †	N/A	207.13 ± 87.35 (185.0)	
Door-to-groin (DGT) †	N/A	75.17 ± 40.74 (73.0)	

Data are *N* (%) for categorical variables or mean ± SD (median) for numerical variables †. Regarding categorical variables, the groups are statistically compared by a chi-square test of independence; for numerical variables, differences in group medians are tested by a Mann-Whitney *t*-test. All *p*-values (two-sided alternative hypothesis) are reported after Benjamini-Hochberg correction; EVT, endovascular therapy; IVT, intravenous thrombolysis; *N*, number of patients; N/A, not applicable; NIHSS, National Institutes of Health Stroke Scale; SD, standard deviation.

**Table 2:** Occurrence of presenting clinical symptoms in anterior circulation stroke (ACS) and posterior circulation stroke (PCS) patients.

Clinical Symptom	Circulation		*p*
	ACS (*N* = 707)	PCS (*N* = 102)	
Limb weakness (HEMIPAR)	636 (89.96)	66 (64.71)	<0.0001
Drooping of the mouth corner (N VII)	588 (83.17)	37 (36.27)	<0.0001
Speech difficulties (SPEECH)	593 (83.88)	61 (59.8)	<0.0001
Sensory impairment (SENSATION)	64 (9.05)	15 (14.71)	0.105
Visual problems (VISION)	5 (0.71)	37 (36.27)	<0.0001
VERTIGO	2 (0.28)	44 (43.14)	<0.0001
HEADACHE	0 (0)	18 (17.65)	N/A
Nausea and/or vomiting (VOMIT)	1 (0.14)	37 (36.27)	N/A
Loss of consciousness (COMA)	9 (1.27)	20 (19.61)	N/A

Data are *N* (%). Symptoms with sufficient frequencies (occurrences) were statistically compared by a chi-square test of independence between ACS and PCS; all *p*-values (two-sided alternative hypothesis) are reported after Benjamini-Hochberg correction; ACS, anterior circulation stroke; *N*, number of patients; N/A, not applicable; PCS, posterior circulation stroke.

**Table 3:** Univariate regression analysis of the dependency of recanalization times.

Explanatory Variable (Predictor)	ODT		ONT		DNT		OGT	DGT
	IVT Only Group (*N* = 398)	EVT (±IVT) Group (*N* = 411)	IVT Only Group (*N* = 398)	EVT (±IVT) Group (*N* = 411)	IVT Only Group (*N* = 398)	EVT (±IVT) Group (*N* = 411)	EVT (±IVT) Group (*N* = 411)	EVT (±IVT) Group (*N* = 411)
Age, (years)	−0.0050 (0.0026); 0.076	−0.0021 (0.0030); 0.8174	−0.0034 (0.0022); 0.125	0.0025 (0.0019); 0.197	6.215 × 10^−5^ (1.558 × 10^−3^); 0.968	0.0060 (0.0028); 0.0312	−0.0004 (0.0018); 0.798	0.0042 (0.0021); 0.0476
Male sex	−0.00007 (0.0661); 0.999	−0.2142 (0.0751); 0.0517	0.0207 (0.0573); 0.718	−0.0428 (0.0484); 0.377	−0.0134 (0.0395); 0.734	0.0314 (0.0676); 0.642	−0.1325 (0.0477); 0.0058	0.0275 (0.0553); 0.619
NIHSS baseline	−0.0270 (0.0065); 0.0006	−0.0111 (0.0068); 0.3363	−0.0174 (0.0057); 0.0027	−0.0033 (0.0049); 0.495	−0.0052 (0.0038); 0.178	0.0045 (0.0063); 0.474	−0.0047 (0.0041); 0.249	0.0026 (0.0047); 0.581
Posterior vascular territory	0.3190 (0.0927); 0.0021	0.2141 (0.1325); 0.3363	0.284 (0.0808); 0.0005	0.1889 (0.0866); 0.0304	0.1063 (0.0579); 0.0672	0.1701 (0.1277); 0.184	0.2232 (0.0791); 0.0051	0.1729 (0.0852); 0.0433
Clinical symptoms								
Limb weakness (HEMIPAR)	−0.1927 (0.0785); 0.0271	−0.0868 (0.1662); 0.8278	−0.1673 (0.0681); 0.0146	−0.1384 (0.1328); 0.298	−0.0655 (0.0479); 0.173	0.0930 (0.1680); 0.58	−0.2052 (0.1081); 0.0585	−0.1231 (0.1217); 0.312
Drooping of the mouth corner (N VII)	−0.1554 (0.0712); 0.0486	−0.1816 (0.1060); 0.3363	−0.1619 (0.0619); 0.0093	−0.1678 (0.0694); 0.0165	−0.0669 (0.0431); 0.122	−0.1952 (0.0976); 0.0469	−0.0674 (0.0640); 0.293	−0.2539 (0.0763); 0.0009
Speech difficulties (SPEECH)	−0.2164 (0.0758); 0.0100	0.0069 (0.1155); 0.9530	−0.2275 (0.0662); 0.0006	−0.0366 (0.0714); 0.609	−0.1063 (0.0471); 0.0247	−0.0525 (0.1000); 0.6	−0.0752 (0.0675); 0.266	−0.099 (0.0767); 0.198
Sensory impairment (SENSATION)	0.0039 (0.0887); 0.9990	0.4925 (0.1955); 0.0909	0.0624 (0.0781); 0.424	−0.0707 (0.1121); 0.529	−0.0197 (0.0538); 0.715	0.0250 (0.2233); 0.911	0.2102 (0.1080); 0.0526	−0.1226 (0.1248); 0.327
Visual problems (VISION)	0.3724 (0.1180); 0.0046	0.2739 (0.2404); 0.5632	0.2694 (0.1039); 0.0099	0.1271 (0.1565); 0.418	0.0840 (0.0757); 0.268	−0.0596 (0.2491); 0.811	0.2008 (0.1466); 0.172	0.2197 (0.1513); 0.148
VERTIGO	0.37064 (0.1037); 0.0019	0.3421 (0.2625); 0.5335	0.3434 (0.0923); 0.0002	−0.0913 (0.1746); 0.602	0.1410 (0.0661); 0.0337	0.2770 (0.2484); 0.266	0.1908 (0.1688); 0.259	0.1165 (0.1744); 0.504
HEADACHE	0.2488 (0.1623); 0.1638	−0.1691 (0.5841); 0.8939	0.238 (0.1447); 0.101	−0.1183 (0.3468); 0.733	0.0359 (0.0970); 0.711	−0.2124 (0.4945); 0.668	−0.3638 (0.4105); 0.376	0.0696 (0.3666); 0.85
Nausea and/or *666* vomiting (VOMIT)	0.4029 (0.1135); 0.0019	0.1496 (0.2408); 0.8278	0.357 (0.1012); 0.0004	0.3517 (0.1547); 0.0241	0.1181 (0.0731); 0.107	0.1613 (0.2041); 0.43	0.0456 (0.1692); 0.787	0.2319 (0.1844); 0.209
Loss of consciousness (COMA)	−0.2572 (0.4077); 0.6252	−0.0876 (0.1662); 0.8278	0.0447 (0.3643); 0.902	0.2309 (0.1237); 0.0636	0.3891 (0.2192); 0.0767	0.4960 (0.1745); 0.0049	0.1814 (0.0992); 0.0685	0.2670 (0.1181); 0.0244
Occlusion								
ICAe	N/A	−0.0803 (0.1091); 0.8174	N/A	−0.0622 (0.0692); 0.37	N/A	−0.0011 (0.0971); 0.991	−0.0035 (0.0713); 0.96	−0.1015 (0.0865); 0.242
ICAi	N/A	−0.0085 (0.0991); 0.9530	N/A	−0.0300 (0.0657); 0.648	N/A	0.0887 (0.0880); 0.315	−0.0481 (0.0657); 0.465	0.0743 (0.0740); 0.315
MCA/M1	N/A	−0.0175 (0.0860); 0.9229	N/A	−0.1082 (0.0554); 0.0524	N/A	−0.0994 (0.0789); 0.209	−0.1132 (0.0533); 0.0346	−0.1206 (0.0608); 0.0481
MCA/M2	N/A	−0.1073 (0.1091); 0.654	N/A	0.0470 (0.0703); 0.504	N/A	0.0536 (0.1000); 0.593	−0.0132 (0.0690); 0.848	0.0660 (0.0815); 0.418
ACA	N/A	−0.3067 (0.2627); 0.5632	N/A	−0.2934 (0.1736); 0.0925	N/A	0.1950 (0.2229); 0.383	−0.1851 (0.1689); 0.274	0.0331 (0.1848); 0.858
PCA	N/A	0.0849 (0.2410); 0.8861	N/A	0.3438 (0.1548); 0.0275	N/A	0.2230 (0.2228); 0.318	0.1866 (0.1565); 0.234	0.2837 (0.1576); 0.0727
VA	N/A	−0.1268 (0.2938); 0.8632	N/A	0.0970 (0.1565); 0.536	N/A	0.4025 (0.2476); 0.106	0.0744 (0.1691); 0.66	0.1508 (0.1656); 0.363
BA	N/A	0.3080 (0.1542); 0.2580	N/A	0.1902 (0.1114); 0.0894	N/A	0.0554 (0.1598); 0.729	0.2578 (0.0916); 0.0052	0.1518 (0.1049); 0.149
IVT	N/A	−0.4410 (0.0798); <0.0001	N/A	N/A	N/A	N/A	−0.0089 (0.0518); 0.863	−0.1019 (0.0572); 0.0757

Outputs from univariate regression models (OLS) for log transformed dependent variable (time intervals) are reported as follows: beta (SE); *p* value; ACA, anterior cerebral artery; BA, basilar artery; DGT, door-to-groin time; DNT, door-to-needle time; EVT, endovascular therapy; iCAe, extracranial internal carotid artery; ICAi, intracranial internal carotid artery; IVT, intravenous thrombolysis; MCA/M1, M1 segment of the middle cerebral artery; MCA/M2, M2 segment of the middle cerebral artery; ***N***, number of patients; N/A, not applicable; NIHSS, National Institutes of Health Stroke Scale; ODT, onset-to-door time; OGT, onset-to-groin time; ONT, onset-to-needle time; PCA, posterior cerebral artery; SE, standard error; VA, vertebral artery.

**Table 4:** Best multivariable models of observed time intervals.

Patient Group	Observed Time Interval (Outcome)	Explanatory Variable (Predictor)	Beta	Standard Error	*p*
IVT only	Onset-to-door time (ODT)	NIHSS	−1.741	0.687	0.0118
		Limb weakness (HEMIPAR)	−12.608	8.161	0.1233
		Speech difficulties (SPEECH)	−18.927	7.619	0.0135
		Nausea and/or vomiting (VOMIT)	31.159	11.748	0.0084
	Onset-to-needle time (ONT)	NIHSS	−1.5934	0.7575	0.036
		Limb weakness (HEMIPAR)	−15.0672	8.9026	0.092
		Speech difficulties (SPEECH)	−24.564	8.4185	0.004
		Nausea and/or vomiting (VOMIT)	43.7237	13.1284	0.001
	Door-to-needle time (DNT)	Speech difficulties (SPEECH)	−5.163	2.737	0.060
		VERTIGO	8.575	3.84	0.026
EVT (±IVT)	Onset-to-needle time (ONT)	Vascular territory—anterior	−24.76	12.99	0.058
		Drooping of the mouth corner (N VII)	−15.76	10.43	0.132
	Door-to-needle time (DNT)	Loss of consciousness (COMA)	22.675	6.046	0.0002
	Onset-to-groin time (OGT)	Vascular territory—anterior	−47.32	16.89	0.005
	Door-to-groin time (DGT)	Drooping of the mouth corner (N VII)	−20.794	6.015	0.0006

**Beta** is the estimated regression coefficient of the multivariable regression model (OLS) that can be interpreted as the population (point) estimate of the difference in the mean time from the reference group (no symptoms, posterior vascular territory). Beta for the NIHSS is the estimate of the marginal (unit) change, i.e., every one-point increase in the NIHSS value is expected to shorten the mean ONT in the IVT group by 1.59 min; EVT, endovascular therapy; IVT, intravenous thrombolysis; NIHSS, National Institutes of Health Stroke Scale.

## Data Availability

The datasets analyzed during the current study are available from the corresponding author on reasonable request.
